# Current Intensity and Immediate Analgesic Effects of Transcutaneous Electrical Nerve Stimulation in Patients with Lumbosacral Pain: A Preliminary Observational Study

**DOI:** 10.3390/healthcare14142121

**Published:** 2026-07-15

**Authors:** Joanna Witkoś, Grzegorz Błażejewski, Galina Mratskova, Ewa Strój-Loranc, Tomasz Kogut, Magdalena Hartman-Petrycka

**Affiliations:** 1 Faculty of Health Sciences, Andrzej Frycz Modrzewski Krakow University, 30-705 Krakow, Poland; 2 Department of Physical and Rehabilitation Medicine, Medical Faculty, Trakia University, 6000 Stara Zagora, Bulgaria; 3 Specialmed Medical Center, 32-410 Dobczyce, Poland; 4 Department of Basic Biomedical Science, Faculty of Pharmaceutical Sciences in Sosnowiec, Medical University of Silesia, 41-200 Katowice, Poland

**Keywords:** transcutaneous electrical nerve stimulation, TENS, pain, lumbosacral pain, stimulation intensity, electrotherapy, analgesia

## Abstract

Background: Transcutaneous electrical nerve stimulation (TENS) is widely used for pain management; however, the optimal stimulation intensity remains uncertain. This preliminary observational study investigated the association between TENS current intensity and immediate analgesic effects in patients with lumbosacral pain. Methods: Fifty-three adults with lumbosacral pain undergoing routine physiotherapy were enrolled. Participants were classified post hoc according to their preferred stimulation intensity into a low-intensity group (L, *n* = 26), receiving stimulation at approximately the sensory threshold level, and a high-intensity group (H, *n* = 27), receiving stimulation at the highest tolerated intensity. TENS was delivered using an ASTAR ETIUS device (100 Hz, 100 μs, biphasic symmetrical waveform) for 20 min. Pain intensity was assessed before and immediately after treatment using a 10-point Visual Analog Scale (VAS). Between-group baseline characteristics were compared using the Mann–Whitney U test. Within-group changes were assessed using the Wilcoxon signed-rank test, and associations between current intensity and pain reduction were evaluated using Spearman’s rank correlation coefficient (rs). Results: Baseline demographic and clinical characteristics did not differ significantly between groups. No significant change in pain intensity was observed in Group L. In contrast, Group H demonstrated a significant reduction in VAS scores following TENS (VAS before Mdn = 5, VAS after Mdn = 4, Z = 4.11, *p* < 0.001, effect size r = 0.88). In this group, higher stimulation intensity was associated with greater pain reduction (r_s_ = −0.48, *p* = 0.011). Conclusions: In this study, TENS delivered at the highest tolerated intensity was associated with greater immediate pain relief than stimulation applied near the sensory threshold. Because of the observational design, post hoc group allocation, and lack of randomization, the findings should be interpreted as exploratory and hypothesis-generating. Further randomized controlled trials are needed to determine whether higher stimulation intensities causally improve TENS analgesic efficacy.

## 1. Introduction

Pain is one of the leading causes of disability worldwide and substantially impairs physical functioning, emotional well-being, and quality of life. Chronic musculoskeletal pain, particularly low back pain, represents a major public health challenge associated with increased healthcare utilization, work absenteeism, and socioeconomic burden [1,2].

Transcutaneous electrical nerve stimulation (TENS) is a non-invasive, non-pharmacological intervention commonly used for the management of acute and chronic pain. Because of its favorable safety profile, low cost, and suitability for self-administration, TENS remains widely used in rehabilitation and physical medicine settings [3,4,5,6]. However, despite more than five decades of clinical application, uncertainty persists regarding its effectiveness and the optimal selection of stimulation parameters. Recent systematic reviews and meta-analyses suggest that TENS can provide clinically meaningful pain relief during or immediately after treatment, although treatment effects vary across studies and clinical populations [7,8,9]. This variability may be partly explained by differences in stimulation dosing, including current intensity, frequency, pulse duration, treatment duration, and electrode placement [10,11].

The analgesic effects of TENS have traditionally been explained by the gate control theory proposed by Melzack and Wall, according to which activation of large-diameter afferent fibers inhibits the transmission of nociceptive signals within the dorsal horn of the spinal cord [12]. Although this concept remains fundamental for understanding the clinical application of TENS, contemporary pain neuroscience indicates that its mechanisms of action are considerably more complex. Current evidence suggests that TENS influences pain processing at the peripheral, spinal, and supraspinal levels of the nervous system. At the spinal level, activation of non-nociceptive afferent fibers may inhibit the transmission of nociceptive input through segmental inhibitory interneurons, thereby reducing the excitability of second-order neurons involved in pain transmission [13,14]. Experimental studies have also demonstrated that TENS activates descending inhibitory pathways originating from supraspinal structures, including the periaqueductal gray, rostroventral medulla, and other brain regions involved in endogenous pain modulation [15,16]. These pathways utilize neurotransmitters such as serotonin, noradrenaline, γ-aminobutyric acid (GABA), and endogenous opioids to suppress nociceptive processing within the central nervous system [17]. Furthermore, increasing attention has been directed toward the role of TENS in reducing central sensitization, a phenomenon characterized by increased responsiveness of central nociceptive neurons that contributes to pain amplification and persistence in many chronic pain conditions [18]. Experimental and clinical studies indicate that appropriately dosed TENS may attenuate hyperalgesia, decrease central excitability, and modulate quantitative sensory testing outcomes, suggesting broader neuromodulatory effects beyond simple segmental inhibition [19]. These observations support the concept that TENS should be viewed not merely as a peripheral sensory intervention but as a treatment capable of influencing multiple levels of pain processing within the nervous system. Importantly, the magnitude of these neurophysiological effects appears to depend on stimulation dosing. Neural recruitment during electrical stimulation is determined not only by current amplitude but also by pulse duration, stimulation frequency, waveform characteristics, electrode size, electrode placement, tissue impedance, and individual patient characteristics [20]. Nevertheless, current intensity remains one of the most clinically relevant parameters because it directly determines the extent of afferent fiber activation and the strength of sensory input delivered to pain-modulating neural networks [16]. Consequently, optimizing stimulation intensity may be considered a clinically relevant strategy for maximizing activation of endogenous pain-modulating systems and improving the analgesic effectiveness of TENS.

Among the adjustable stimulation parameters, current intensity is increasingly recognized as a critical determinant of TENS effectiveness. Previous experimental studies demonstrated that stronger, yet comfortable, stimulation produces greater hypoalgesic effects than stimulation delivered at or below the sensory threshold [16,17,18]. Similarly, recent clinical studies and meta-analyses have suggested that inadequate stimulation intensity may contribute to inconsistent therapeutic outcomes reported in the literature [7,10,20]. Nevertheless, despite growing recognition of the importance of stimulation intensity, relatively little is known about how patient-selected intensity levels influence immediate analgesic outcomes during routine clinical practice. Most previous investigations have been conducted under experimental conditions or within highly standardized research protocols, which may not fully reflect everyday physiotherapy settings. Therefore, an important knowledge gap remains regarding the relationship between preferred or tolerated TENS current intensity and immediate pain relief in patients receiving routine treatment for lumbosacral pain. Understanding this relationship may help optimize TENS dosing strategies and improve clinical effectiveness.

The aim of this study was to investigate the association between TENS current intensity and immediate analgesic response in patients with lumbosacral pain. We hypothesized that participants who preferred and tolerated higher stimulation intensities would experience greater immediate reductions in pain intensity than those receiving stimulation near the sensory threshold.

## 2. Materials and Methods

### 2.1. Study Design and Settings

This was a preliminary observational study conducted in a routine clinical physiotherapy setting. The study was designed to explore the association between patient-preferred TENS current intensity and immediate pain reduction after a single treatment session in adults with lumbosacral pain. Participants were classified post hoc according to the stimulation intensity selected and tolerated during the routine TENS procedure. The study was conducted at the Municipal Center for Medical Services in Lisia Góra, Lesser Poland Voivodeship, Poland. Recruitment began on 3 February 2025 and was completed on 28 July 2025. The study was conducted in accordance with the Declaration of Helsinki and was approved by the Institutional Ethics Committee of Andrzej Frycz Modrzewski Krakow University.

### 2.2. Participants

This study included 53 adults aged 35–77 years (mean age: 55.0 ± 12.2 years), comprising 34 women and 19 men. Participants were recruited from patients attending the Municipal Center for Medical Services in Lisia Góra, who had been referred by a physician for physiotherapy because of pain localized in the lumbosacral region of the spine. Participants were eligible for inclusion if they were aged 18 years or older, reported lumbosacral pain at the time of treatment, had been referred for TENS therapy as part of routine physiotherapy management, were able to understand and complete the Visual Analog Scale (VAS), and voluntarily agreed to participate in the study. On the study day, no other physical medicine modalities were administered before or during the assessment apart from the TENS session evaluated in the present study. Pain intensity was assessed immediately before and immediately after this single TENS application to capture its immediate analgesic response. Following the assessed TENS session, participants proceeded with their routine rehabilitation program, which could include therapeutic exercises delivered as part of usual clinical care.

Participants were excluded if they did not provide informed consent or presented with contraindications to electrotherapy. These included the presence of an implanted cardiac pacemaker or other implanted electronic device, pregnancy, epilepsy, active malignancy in the treatment area, impaired skin integrity at the electrode placement site, severe sensory disturbances in the lumbosacral region, acute infection, fever, or any other condition considered by the referring physician to contraindicate electrotherapy. Because analgesic medications may influence pain perception and potentially modify responses to TENS [21], participants were instructed to avoid taking analgesic medication before the study session. However, actual analgesic consumption prior to treatment was not formally recorded or verified. Therefore, the potential influence of analgesic use on the observed pain responses cannot be completely excluded and should be considered when interpreting the findings.

All participants received standardized information regarding the purpose and procedure of the study before providing verbal informed consent. Data were collected and analyzed anonymously. Because this was a preliminary exploratory observational study, no formal a priori sample size calculation was performed. The analyzed sample consisted of all eligible participants recruited during the study period. Baseline demographic and anthropometric characteristics of participants in Groups L and H are presented in Table 1. No statistically significant differences were observed between the groups with respect to baseline demographic and anthropometric characteristics. Effect sizes were negligible to small (r = 0.02–0.17), indicating good baseline comparability between the groups.

### 2.3. TENS Procedure and Outcome Measures

**Figure 1:**
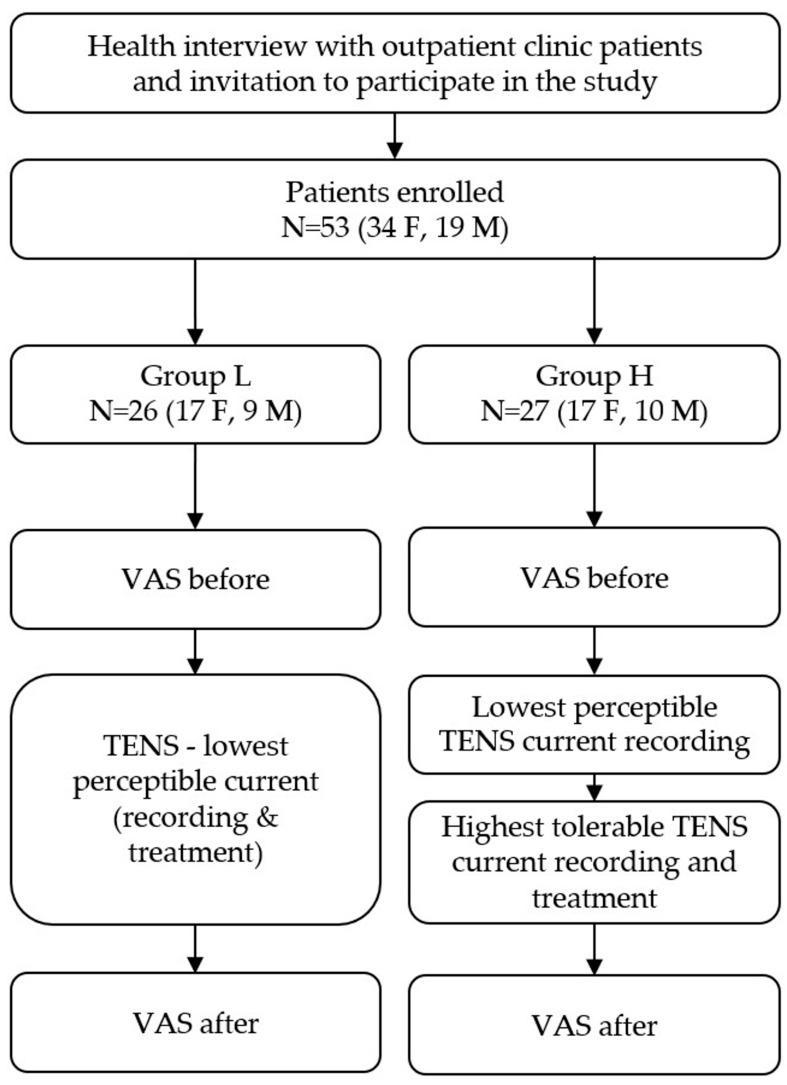
Flow diagram of the study design and TENS assessment procedure.

The study involved the assessment of pain intensity using the Visual Analog Scale (VAS) and the evaluation of its immediate change following a single TENS session (Figure 1). The VAS is a widely used instrument in clinical practice for assessing pain intensity and monitoring the effectiveness of pain management interventions. Participants rated their pain on a 10-point scale, where 0 represented “no pain” and 10 represented “the worst pain imaginable.”

TENS was delivered during a routine physiotherapy session using the ASTAR ETIUS device (Astar, Bielsko-Biała, Poland). Stimulation parameters were standardized for all participants and consisted of a frequency of 100 Hz, pulse duration of 100 μs, and a biphasic symmetrical waveform [14]. The duration of the TENS session was 20 min. Electrodes were positioned bilaterally in the paraspinal lumbosacral region corresponding to the area of greatest pain indicated by the participant. A single stimulation channel was used. Two silicone–carbon rubber electrodes measuring 7.5 × 9 cm were applied in moistened sponge covers soaked in lukewarm water. The inter-electrode distance was approximately 15 cm. Electrode placement was standardized across participants and performed according to routine clinical practice.

Before stimulation, pain intensity was assessed using the VAS. The sensory threshold was determined by gradually increasing the current intensity until the participant first perceived the stimulation. The current amplitude was then further increased according to the participant’s preferred and tolerated level of stimulation. Group L (low-intensity group) included participants who preferred stimulation perceived as a mild sensory stimulus close to the sensory threshold. Group H (high-intensity group) included participants who preferred stimulation at the highest tolerated, yet non-painful, intensity. The investigators did not influence the selection of stimulation intensity and were limited to recording the treatment parameters and outcome measures. Immediately after completion of the TENS session, pain intensity was reassessed using the VAS.

### 2.4. Statistical Analyses

The database was created in Microsoft Excel 2019 (Microsoft Corporation, Redmond, WA, USA) and subsequently imported into Statistica 13.3 (TIBCO Software Inc., Palo Alto, CA, USA) for statistical analyses. The distribution of variables in Groups L and H was assessed using histograms and the Shapiro–Wilk test. Since the assumption of normality was not met for some variables, nonparametric tests were applied in further analyses, including the Mann–Whitney U test, the Wilcoxon signed-rank test, and Spearman’s rho correlation coefficient. Between-group baseline characteristics were compared using the Mann–Whitney U test. Within-group changes were assessed using the Wilcoxon signed-rank test, and associations between current intensity and pain reduction were evaluated using Spearman’s rank correlation coefficient (rs).

The following variables were analyzed:

Basic participant characteristics, including age [years], height [cm], body weight [kg], and BMI [kg/m^2^] in Groups L and H (the Mann–Whitney U test).

The lowest TENS current intensity perceived by participants in Groups L and H [mA] (the Mann–Whitney U test).

Pain intensity measured with the VAS before and after the TENS session in Groups L and H (the Wilcoxon signed-rank test).

The association between change in pain intensity (VAS after − VAS before) and the highest current intensity applied in Group H [mA] (Spearman’s rank correlation coefficient, rs).

Results were considered statistically significant at *p* < 0.05.

## 3. Results

**Figure 2:**
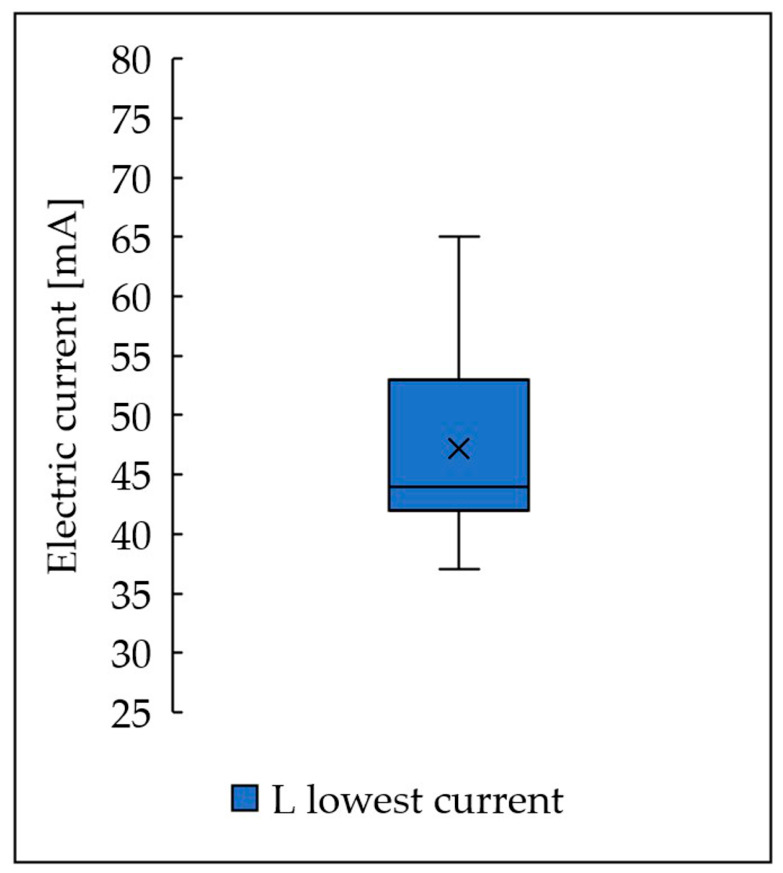
The lowest current intensities in group L. Median, Box–Interquartile range, Mustash–Min_ Max, x–Mean.

**Figure 3:**
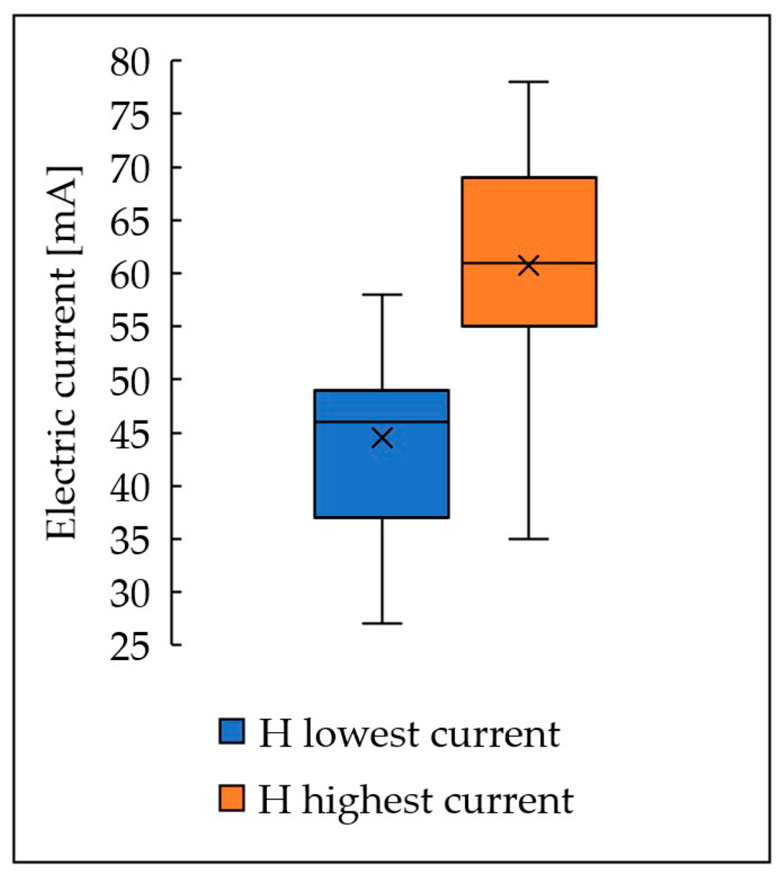
The lowest and highest current intensities in group H. Median, Box–Interquartile range, Mustash–Min_ Max, x–Mean.

In Groups L and H, the lowest TENS current intensity perceived by participants did not differ significantly (*p* = 0.748, r = 0.04) (Figure 2 and Figure 3). In Group L, the median of the lowest perceptible current intensity was 44 mA, with lower and upper quartile values of 42 mA and 53 mA, respectively. In Group H, the median of the lowest perceptible current intensity was 46 mA, with quartile values of 37 mA (lower) and 49 mA (upper). This finding indicates comparable sensory thresholds in both groups. The median of the highest current intensity applied during the procedure in Group H was 61 mA, with lower and upper quartile values of 55 mA and 69 mA, respectively (Figure 3).

**Figure 4:**
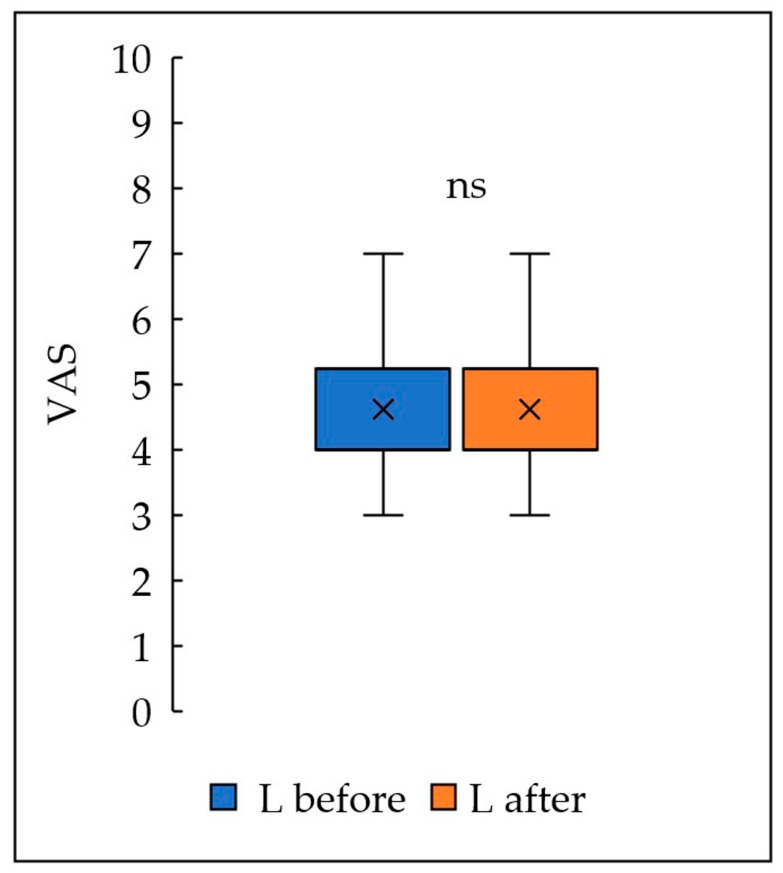
Pain intensity assessed using the VAS (0–no pain, 10–worst imaginable pain) in group L before and after the TENS session. ns–not significant; Median, Box–Interquartile range, Mustash–Min_ Max, x–Mean.

**Figure 5:**
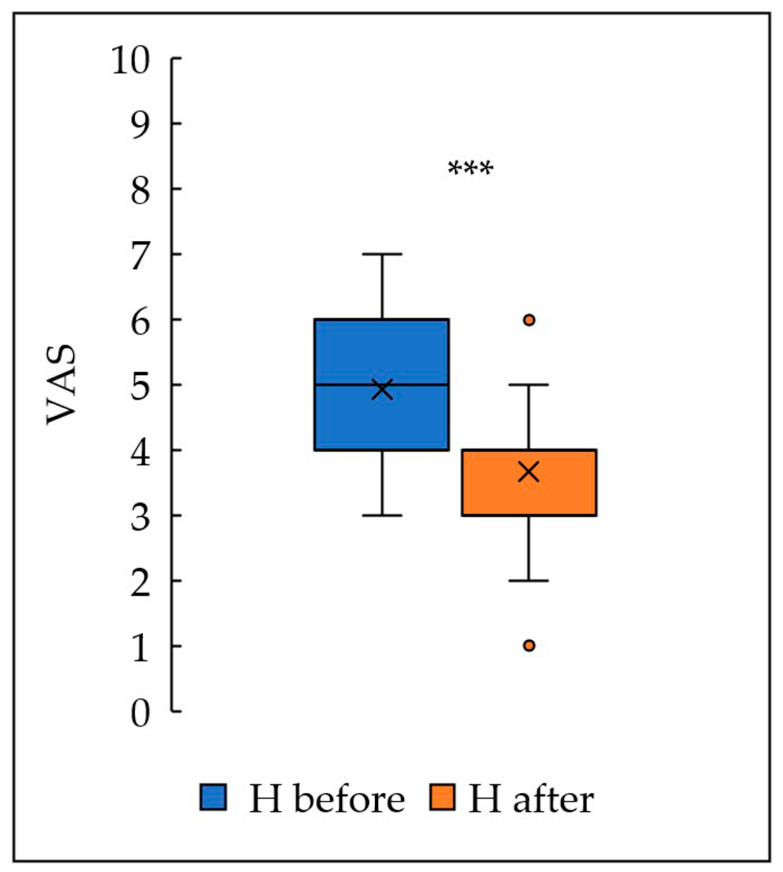
Pain intensity assessed using the VAS (0–no pain, 10–worst imaginable pain) in group H before and after the TENS session. *** *p* < 0.001. Median, Box–Interquartile range, Mustash–Min_ Max, x–Mean.

After the TENS session in Group L, in which participants preferred stimulation within the sensory-threshold range, no changes were observed in pain intensity assessed with the VAS (Figure 4). For all participants, post-treatment pain intensity remained the same as before the intervention, with a median of 4, a lower quartile of 4, and an upper quartile of 5. In Group H, in which participants preferred stimulation up to the highest acceptable current intensity, a statistically significant reduction in VAS pain scores was observed (*p* < 0.001) (Figure 5). Compared with pre-treatment values, the median decreased by 1 point, the lower quartile by 1 point, and the upper quartile by 2 points on the VAS. The magnitude of the observed reduction was large (Wilcoxon signed-rank test: Z = 4.11, *p* < 0.001, effect size r = 0.88), indicating a substantial immediate analgesic effect of high-intensity TENS.

**Figure 6:**
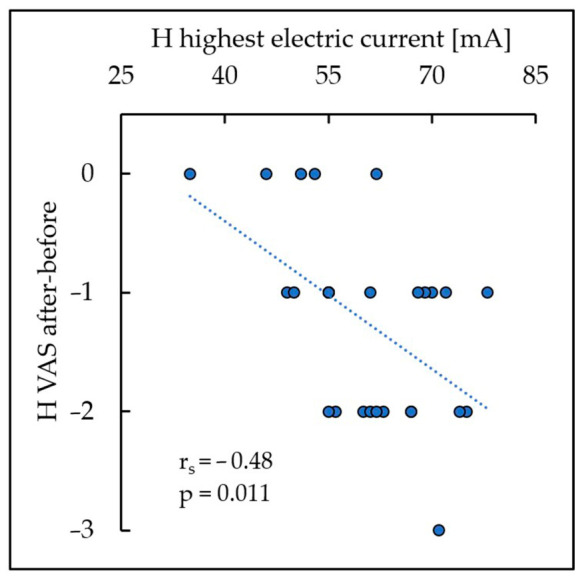
Correlation between pain reduction on the VAS (difference: H VAS after − before) and the applied current intensity in Group H.

In Group L, TENS delivered within the participant-preferred sensory-threshold range, regardless of its absolute current value, did not result in any change in perceived pain intensity. In Group H, in which participants preferred the highest acceptable current intensity was set at the highest level acceptable to the participants, a reduction in VAS pain scores was observed in 1 participant (3.7%) by 3 points, in 10 participants (37.0%) by 2 points, and in 11 participants (40.7%) by 1 point, while 5 participants (18.5%) reported no change in pain intensity (Figure 6). Correlation analysis between pain reduction on the VAS (difference: H VAS after − before) and the highest current intensity applied in Group H (H highest current) revealed a statistically significant association (r_s_ = −0.48; *p* = 0.011).

## 4. Discussion

The present study investigated the relationship between TENS current intensity and immediate analgesic response in patients with lumbosacral pain. The principal finding was that participants who preferred and tolerated higher stimulation intensities experienced significantly greater reductions in pain intensity following a single TENS session than participants receiving stimulation close to the sensory threshold. Furthermore, a significant negative correlation was observed between stimulation intensity and post-treatment pain scores within the high-intensity group, suggesting that higher tolerated current amplitudes were associated with greater immediate pain relief.

The effectiveness of TENS depends on the appropriate selection of stimulation parameters, including stimulation frequency, pulse duration, treatment duration, electrode placement, and current intensity. Among these parameters, stimulation intensity has increasingly been recognized as one of the most clinically relevant factors influencing analgesic outcomes. Previous studies [7,10,15,20] have demonstrated that stimulation delivered at strong but comfortable sensory levels is generally associated with greater hypoalgesic effects than stimulation delivered at or near sensory threshold levels. The findings of the present study are consistent with this concept and support the hypothesis that stimulation intensity may represent an important component of TENS dosing.

Our observations are in agreement with the work of Vance et al. [15], who emphasized that the analgesic effectiveness of TENS is optimized when stimulation is delivered at the highest comfortable intensity tolerated by the patient. Similarly, Johnson et al. [22], in the large meta-TENS analysis, reported moderate-certainty evidence supporting pain reduction during or immediately after TENS treatment and highlighted the importance of adequate stimulation dosing. Although the populations and treatment protocols included in these studies differed from those investigated in the present work, the overall direction of findings appears consistent. Collectively, these data suggest that insufficient stimulation intensity may contribute to variability in treatment outcomes and may partly explain inconsistencies observed across previous TENS studies. Collectively, these data suggest that insufficient stimulation intensity may contribute to variability in treatment outcomes and may partly explain inconsistencies observed across previous TENS studies. This interpretation is also supported by Gibson et al. [2], who concluded in their overview of Cochrane Reviews that although evidence regarding TENS effectiveness remains heterogeneous, TENS may provide clinically meaningful pain relief in selected patient populations when appropriately applied. More recently, Viderman et al. [23] reported in a systematic review and meta-analysis that TENS may contribute to reductions in acute postoperative pain and improve selected postoperative outcomes, further supporting the broader role of TENS as a non-pharmacological analgesic intervention.

Experimental studies also provide support for the present findings. Moran et al. [20] demonstrated that stimulation intensity influences hypoalgesic responses, with stronger stimulation producing greater increases in pressure pain thresholds than low-intensity stimulation. Similar observations have been reported by Delkhoush et al. [24] in patients with knee osteoarthritis, where higher stimulation amplitudes were associated with greater analgesic benefits. Evidence from randomized controlled trials further supports the clinical relevance of stimulation intensity. Ögren et al. [25] demonstrated that high-frequency, high-intensity TENS provided effective postoperative pain relief following laparoscopic cholecystectomy, supporting the concept that stronger stimulation may enhance analgesic outcomes in clinical settings. Furthermore, Pantaleão et al. [26] demonstrated that maintaining or increasing stimulation intensity during treatment sessions may enhance analgesic responses, suggesting that adaptation to stimulation should be considered when optimizing TENS treatment protocols. Although these studies differed in methodology and patient populations, they collectively support the concept of an intensity-dependent analgesic response.

The observed association between higher stimulation intensity and greater pain reduction may reflect more effective activation of endogenous pain-modulating systems. Contemporary neurophysiological models suggest that stronger but comfortable TENS may produce greater engagement of inhibitory mechanisms involved in pain processing [15,16,17]. Increasing evidence also suggests that appropriately dosed TENS may influence central sensitization and hyperalgesia, both of which play important roles in the persistence of chronic musculoskeletal pain. Recent reviews by Amer-Cuenca et al. [27] and DeJesus et al. [10] have highlighted the potential neuromodulatory effects of TENS and emphasized that stimulation intensity may influence the magnitude of these responses. Stronger but comfortable stimulation may therefore contribute to more pronounced modulation of pain processing. However, because neurophysiological outcomes were not directly measured in the present study, these explanations should be regarded as biologically plausible mechanisms supported by previous literature rather than direct evidence derived from our findings.

The clinical implications of the present results are noteworthy. In routine physiotherapy practice, current intensity is often adjusted primarily according to patient comfort. The present findings suggest that clinicians should consider achieving the highest comfortable and well-tolerated stimulation intensity rather than maintaining stimulation close to sensory threshold levels. Such an approach may enhance the immediate analgesic response while maintaining patient comfort and treatment adherence. Furthermore, Martins-de-Sousa et al. [28] demonstrated that TENS may provide additional benefits when combined with therapeutic exercise in patients with chronic musculoskeletal pain, highlighting the potential value of optimizing stimulation parameters within multimodal rehabilitation programs. Nevertheless, the optimal balance between treatment efficacy and tolerability remains an important clinical consideration that warrants further investigation.

At the same time, the findings should be interpreted cautiously. The study was observational in nature and participants were classified post hoc according to their preferred stimulation intensity. Consequently, the observed differences cannot be interpreted as evidence of causality. It is possible that unmeasured factors, including psychological characteristics, pain-related beliefs, pain chronicity, individual sensory sensitivity, or other patient-specific variables, influenced both the preferred stimulation intensity and the magnitude of pain reduction observed after treatment. Therefore, the present results should be interpreted primarily as evidence of an association between stimulation intensity and immediate analgesic response.

### 4.1. Limitations

Several limitations should be considered when interpreting the findings of this study. First, the observational design, lack of randomization, absence of a sham-control condition, and post hoc classification of participants according to preferred stimulation intensity preclude causal inference. Second, the sample size was relatively small and participants were recruited from a single clinical center, which may limit generalizability. Third, only the immediate effects of a single 20 min TENS session were assessed, without evaluation of longer-term outcomes. An additional limitation is that the study was conducted in a routine outpatient physiotherapy setting. Although participants continued their usual rehabilitation program after the assessed TENS session, which could include therapeutic exercises, TENS was administered as the first physiotherapy procedure on the study day, and pain intensity was assessed immediately before and immediately after this single TENS application. Therefore, the observed therapeutic effect may reasonably be interpreted as an individual immediate response to TENS rather than as the effect of the entire physiotherapy program delivered on that day. Another important limitation concerns analgesic use. Although participants were instructed to avoid taking analgesic medication before the TENS procedure, actual pre-treatment analgesic consumption was not formally recorded or verified. Because analgesics may influence pain perception and potentially modify responses to TENS, their role as a confounding factor cannot be excluded and may have affected the observed immediate analgesic responses. Finally, although baseline pain intensity did not differ significantly between groups, even small baseline differences may have influenced the magnitude of observed pain reduction.

### 4.2. Future Directions

Future studies should investigate the relationship between TENS intensity and analgesic outcomes using randomized controlled designs with sham stimulation and longer follow-up periods. It would also be valuable to examine repeated treatment sessions, different pain conditions, and the potential influence of analgesic medication use, pain duration, and psychosocial factors on treatment response. Such studies may help establish evidence-based recommendations for optimizing TENS dosing in clinical practice.

## 5. Conclusions

In this observational study, higher tolerated TENS current intensity was associated with greater immediate pain reduction in patients with lumbosacral pain. Participants who preferred stimulation delivered at the highest comfortable intensity experienced more pronounced analgesic responses than those receiving stimulation near the sensory threshold. These findings suggest that stimulation intensity may be an important factor related to the immediate response to TENS and should be considered in future studies on treatment optimization. However, given the observational design and study limitations, the present results should be interpreted as exploratory and hypothesis-generating rather than as definitive evidence of treatment efficacy.

## Figures and Tables

**Table 1:** Characteristics of the low-intensity (L; *n* = 26) and high-intensity (H; *n* = 27) groups.

	Group	Mdn	Q1	Q3	Mean	SD	Min	Max	*p*	Effect Size (r)
Age [year]	L	56.5	44.0	64.0	55.0	12.2	35.0	77.0	0.873	0.022
	H	55.0	44.0	64.0	54.3	12.5	35.0	75.0		
Height [cm]	L	170.0	167.0	175.0	170.2	5.6	160.0	182.0	0.707	0.051
	H	170.0	166.0	172.0	169.6	4.4	163.0	180.0		
Weight [kg]	L	76.0	74.0	80.0	76.6	6.3	64.0	88.0	0.218	0.169
	H	76.0	68.0	80.0	73.9	6.9	62.0	85.0		
BMI [kg/m^2^]	L	26.2	25.0	26.9	26.5	2.3	22.9	34.4	0.722	0.049
	H	26.4	24.6	27.0	25.7	2.0	20.8	28.7		

n—number, Mdn—median, Q1—first quartile, Q3—third quartile, SD—standard deviation, Min—minimum, Max—maximum.

## Data Availability

The raw data supporting the conclusions of this article will be made available by the authors on request.

## Abbreviations

The following abbreviations are used in this manuscript:

TENS	Transcutaneous electrical nerve stimulation
VAS	Visual Analog Scale
BMI	Body mass index
